# An integrative model of pro- and anti-inflammatory signaling pathways in macrophage differentiation: the role of NF-*κ*B and CREB

**DOI:** 10.3389/fimmu.2025.1639005

**Published:** 2026-01-02

**Authors:** David Martínez-Méndez, Ilean Z. Aguilar-Elguea, Lilian S. Castelán-Pacheco, Luis Armando Jiménez-Alvarez, Alfredo Cruz-Lagunas, Joaquín Zúñiga, Carlos Villarreal, Leonor Huerta

**Affiliations:** 1 Instituto de Investigaciones Biomédicas, Departamento de Inmunología, Universidad Nacional Autónoma de México, Mexico City, Mexico; 2 Instituto de Física, Universidad Nacional Autónoma de México, Mexico City, Mexico; 3 Instituto Nacional de Enfermedades Respiratorias Ismael Cosío Villegas, Secretaría de Salud, Tecnológico de Monterrey, Escuela de Medicina y Ciencias de la Salud, CCM, Mexico City, Mexico

**Keywords:** inflammation, macrophage differentiation, anti-inflammatory cytokines, CREB, biological network, LPS, IFN-γ, mathematical model

## Abstract

**Background:**

Monocytes are essential players of the innate immune response and adapt their functional states in response to different antigenic and cytokine environments. Integrating the complexity of monocyte intracellular signaling into a mathematical model can support the understanding of dynamic transitions that are crucial for immune regulation.

**Objective:**

To formulate a comprehensive mathematical model of monocyte activation, differentiation, and metabolic adaptation dynamics in response to a variety of stimulus and cytokine microenvironment.

**Methodology:**

The model comprises a 128-node complex regulatory network based on known components of monocyte activation signal pathways. Node interactions are described by continuous fuzzy logic rules, and includes signaling events induced by LPS, activating IgG immune complexes, ssRNA, and the IFN-*γ*, IL-4 and IL-10 cytokines. Autocrine feedback loops for IL-10 and TNF-*α*, and a metabolism subnetwork were included. The network was analyzed by a set of ordinary differential equations (ODEs) system. The system outputs describe the dynamics of cell metabolic activity, activation of transcription factors, cytokine production and phagocytosis. An interactive program was developed as a tool to test the dynamical expression of the monocyte features under different initial conditions (see the https://grci.mx/modelos.html website).

**Results:**

The model captures the dynamics of the main events rendering stable states corresponding to the M1, M2, M2b and M2c macrophage profiles. Results are compatible with the predominance of glycolysis in the M1 and M2b, and oxidative phosphorylation in the M2a and M2c responses. The model shows the convergence to the activation of the NF-*κ*B transcription factor in the pro-inflammatory response, while anti-inflammatory profiles are related to the induction of CREB1, a NF-*κ*B inhibitor and promoter of IL-10 synthesis. Modelling supports a fundamental role of the Akt isoforms Akt1 and Akt3 in the induction of the activity the CREB1 inhibitor GSK3*β* upon IFN-*γ* signaling, so enabling the pro-inflammatory response. The anti-NF-*κ*B activity of IL-4 signaling can turn the response into an M2 profile. The model predicts the relative levels of IFN-*γ* necessary to sustain the inflammatory response. Stochastic modelling proved the robustness of the macrophage differentiation process.

**Conclusion:**

The complex network approach presented here integrates diverse cytokine and antigenic signaling leading to macrophage responses. It supports a mechanism for the IFN-*γ* mediated inhibition of CREB in the balance between pro-inflammatory and anti-inflammatory signals.

## 1 Introduction

Monocytes are involved in pathogen clearance, inflammation, tissue repair, and immune regulation. They pose a variety of receptors for the recognition, internalization, and processing of foreign antigens, along with cytokine receptors that contribute to modulation of adaptive immunity and tissue homeostasis (1, 2). Macrophages differentiate into the M1 phenotype, which is pro-inflammatory and effective in removing pathogens, and three different kind of M2 phenotypes involved in tissue repair and immune regulation. Prototypic M1 macrophages are induced by the combination of lipopolysaccharides (LPS) and interferon gamma (IFN-*γ*), and produce cytokines such as tumor necrosis factor-*α* (TNF-*α*) and interleukine-12 (IL-12). In contrast, M2 macrophages are induced by signals such as interleukine-4 (IL-4) and interleukine-10 (IL-10), which promote tissue repair functions and down-regulate pro-inflammatory responses, preventing excessive tissue damage (3, 4). The M2a phenotype develops in the presence of IL-4, M2b requires IL-1-*β* and additional stimulation, while M2c depends on IL-10 or TGF-*β* (5, 6). Metabolic adaptability associated to M1- or M2-type responses is driven by signaling pathways related to mTOR and AMPK. (7), with glycolysis and oxidative phosphorylation (OXPHOS) modulated in response to energy demands and conditions such as hypoxia and nutrient-deprived environments, commonly encountered in inflamed tissues (8). Thus, macrophage differentiation into alternative phenotypes is determined by the interaction between signals from receptor activation and the cytokine microenvironment. (9).

The inherent complexity of these highly intertwined processes makes it very difficult to understand emergent behaviors, such as phenotype differentiation and immune cell decisions, using only intuition (10). Mathematical analysis based on complex regulatory networks provide an operating way to represent biological circuits composed of numerous elements that contain signaling cascades or switching modules; this approach may yield meaningful qualitative information on the basic topology of relations that determine alternative cell fates, so that it can be used for network analysis without requiring explicit values of biological parameters (11–14). Here, we used this tool to explore how monocytes respond to signals in different microenvironments of cytokines, offering an integrated view of monocyte function. Early studies have been performed by Palma et al. to characterize the logical relationships among genes driving macrophage polarization to the M1 and M2 phenotypes (9).

In this work we put forth a network including signaling from Toll-like receptors (TLR) which recognize pathogen-associated molecular patterns (PAMPs), such as LPS and nucleic acids, detecting bacterial, viral, or fungal infections. The range of TLR includes TLR2 to TLR4, and TLR7 to TLR9, each responding to specific pathogen structures and triggering downstream signaling pathways that activate nuclear factor-kappa B (RelA(p65)/p50 complex)), mitogen-activated protein kinases (MAPKs), and interferon regulatory factors (IRFs) (15, 16). Notably, the cAMP responsive element binding protein (CREB1, or CREB11), which has been considered a pro-inflammatory co-regulator of NF-*κ*B and AP-1 (c-Fos and c-Jun) (17, 18), is also known to play a central role in the inhibition of NF-*κ*B and the production of the anti-inflammatory cytokine IL-10. The activity of the CREB1 inhibitor GSK3*β*, which is constitutively expressed in nonactivated monocytes (19), was introduced in the network to analyze its role in the modulation of the inflammatory response.

## 2 Materials and methods

### 2.1 Regulatory network defined by logical interaction rules

**Figure 1:**
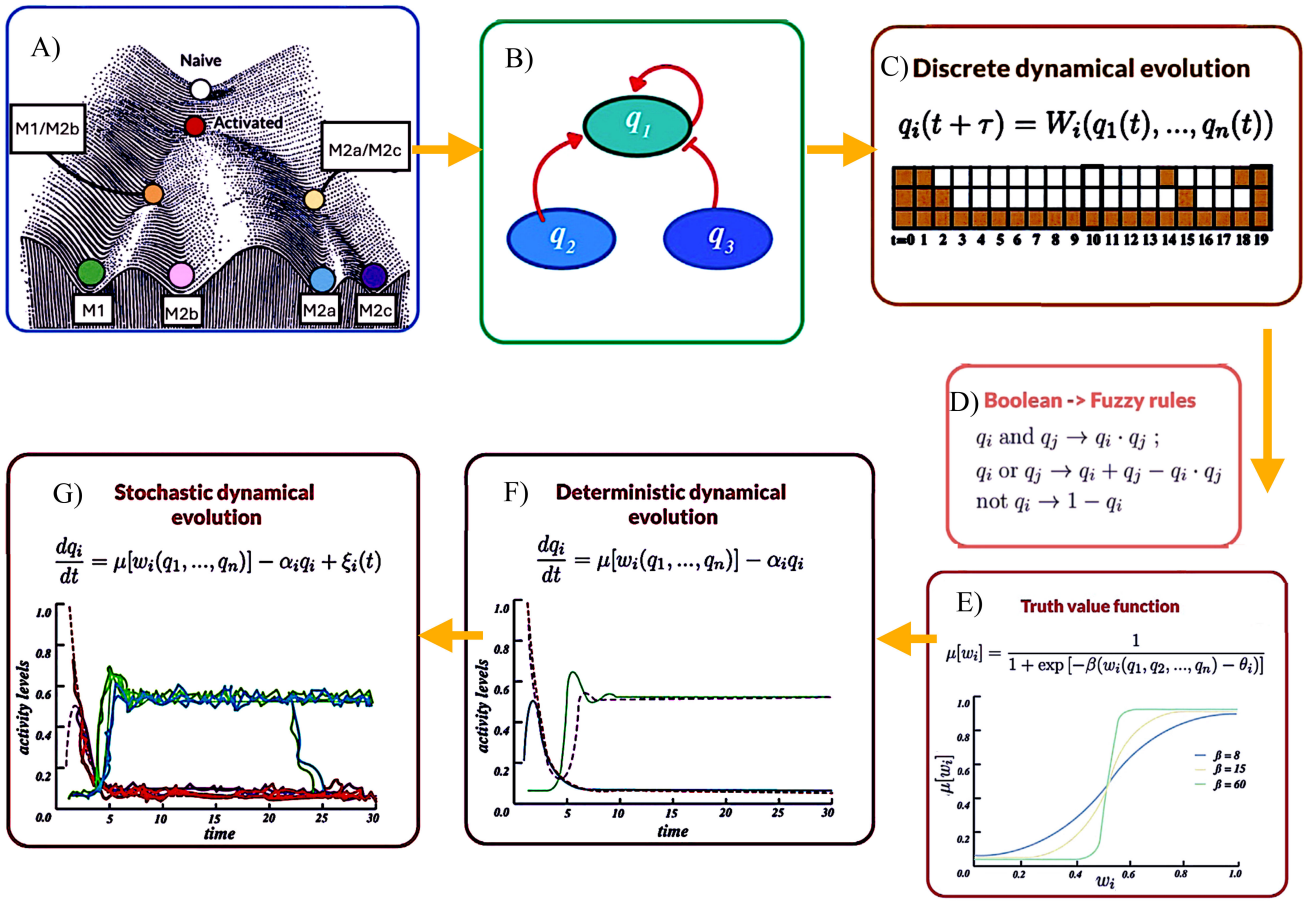
Flux diagram of the mathematical steps leading to the network continuous modelling of macrophage differentiation. **(A)** Epigenetic landscape showing stable states **(Naive, Activated, M1, M2a, M2b, and M2c)** and including hybrid phenotypes. **(B)** Diagram of intracellular signaling as node interactions. **(C)** Discrete dynamical evolution of a network described by Boolean logical rules. **(D)** Conversion from Boolean to fuzzy rules. **(E)** Graph of the truth value function with different node expression rates (beta). **(F)** Deterministic dynamical evolution showing node activity levels over time. **(G)** Robustness analysis can be performed by introducing stochastic perturbations in the network dynamics and calculating differentiation efficiency.

**Figure 2:**
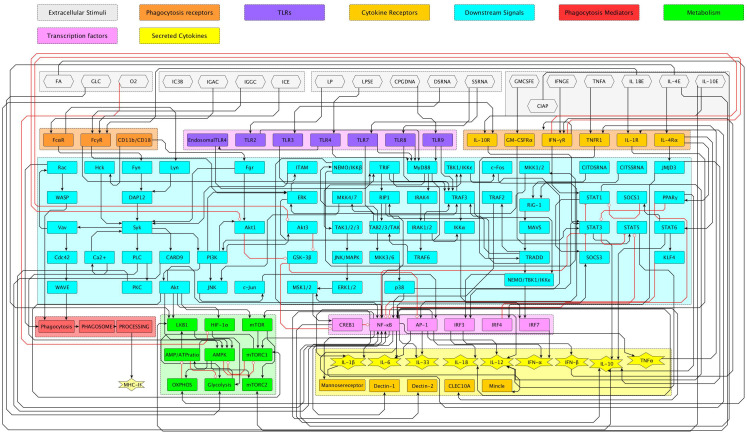
Flow chart illustrating biological signaling pathways, categorized by function using color-coded boxes. Key elements include extracellular stimuli, receptors, extracellular and secreted cytokines, transcription factors, and metabolic processes. Arrows indicate positive (black) and negative (red) interactions. Particular interactions can be visualized in the interactive figure MONOCYTE NET.graphml in the *https://grci.mx* website. Subnetworks for NF-*κ*B, CREB1, AMPK, IL-4 and IL-10 are shown in **Figures 3**, **4**.

The mathematical methodology employed in this work is summarized in **Figure 1**. An overall assumption is that activation, differentiation and effector processes in cells comply with logical rules comprising a regulatory network of node interactions, underlying a (metaphorical) epigenetic landscape (20). **Figure 2** displays the regulatory network for macrophage differentiation put forth in this work. It includes 128 components that incorporate current experimental evidence of the molecular interactions involved in the differentiation of the macrophage phenotypes M1, M2a, M2b, and M2c. The network consists of inputs (environmental stimulating agents) and nodes (representing receptors, enzymes, second messengers, transcription factors, genes, etc.) linked through activating or inhibitory interactions.

**Figure 3:**
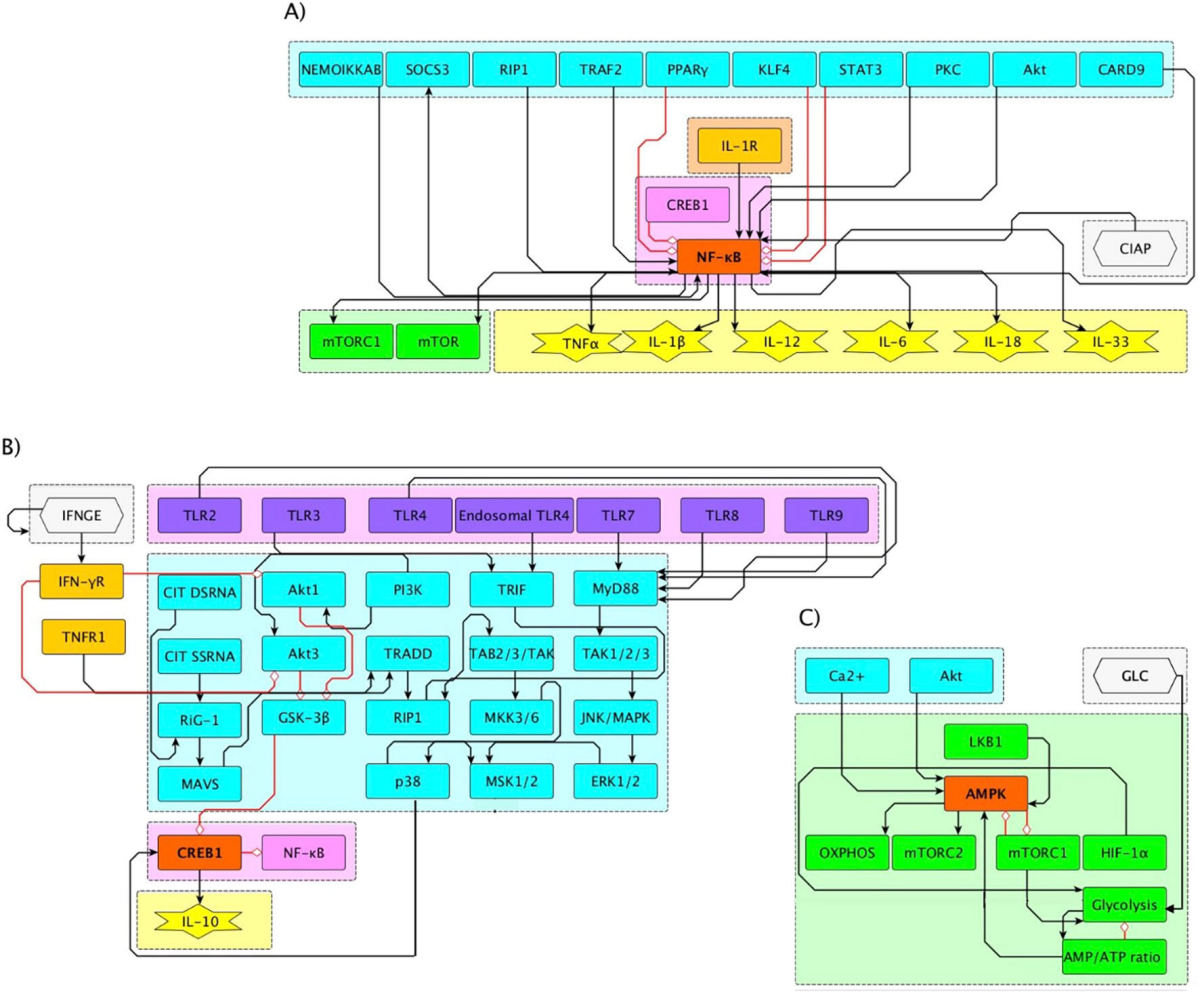
**(A)** Interaction subnetworks of NF-κB, CREB1, and AMPK. **(A)** Stimulatory and inhibitory pathways converge on NFκb activity. NFκb activation leads to secretion of cytokines and activation of the glycolysis-inducer mTORC1. **(B)** CREB1 is activated by TNFαR and cytoplasmic ssRNA and dsRNA via p38; IFN-γ-driven inhibition of Akt1 and Akt3 allows GSK3β to inhibit CREB, leading to NF-κB activation. **(C)** AMPK senses the AMP/ATP ratio and have a negative feedback interaction with mTORC1, defining a switch between oxidative OXPHOS and glycolysis.

**Figure 4:**
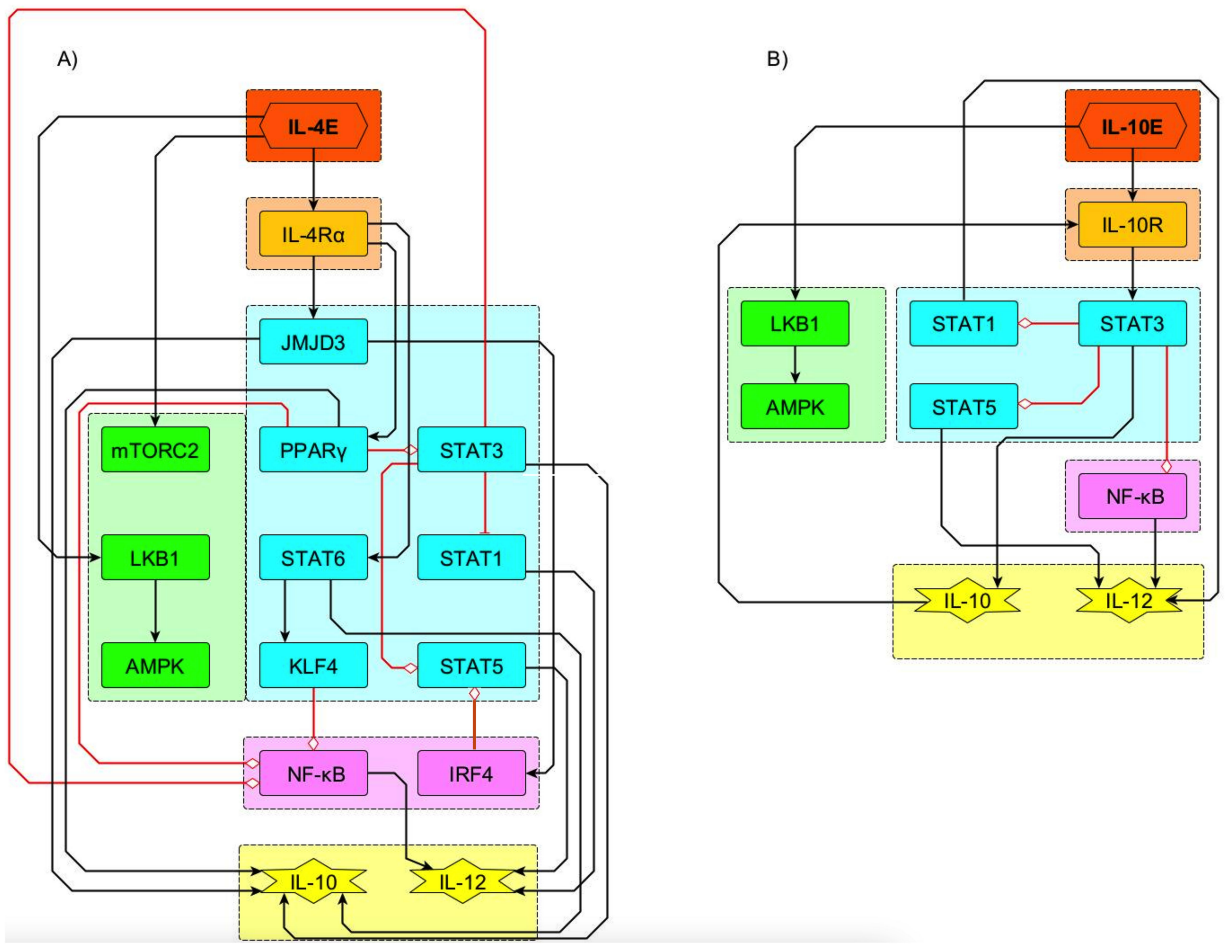
Subnetworks for the IL-4 and IL-10 immunomodulatory effect. Signaling from IL-4 **(A)** and IL-10 **(B)** receptors ((IL-4Rα) and IL-10R, respectively) leads to inhibition of NFkB, production of IL-10 and activation of AMPK. AMPK directs the macrophage metabolism towards oxidative phosphorylation.

In a first level, the network interactions are described by Boolean rules that allow to set up the network architecture, including feedback and switching modules. In the Boolean scheme, logic rules are defined by dichotomous variables with truth values, 0 (unexpressed), or 1 (expressed), and the network dynamics is described in terms of discrete-time mappings whose steady states (attractors) define cell phenotypes. A Boolean approach is fundamental for building the set of regulatory interactions; however, this procedure may be insufficient to account for the complex phenomenology observed in specific experimental studies. A more suitable modelling of biological systems may be achieved by introducing a continuous logical analysis (21, 22). In this approach, the expression levels and concentrations of the signaling elements can acquire any value within a continuous range limited only by functionality constraints and concomitant continuous dynamics. Then, we extended our initial analysis to consider a regulatory network characterized by Fuzzy Logic, aimed to provide formal foundation to logical inferences involving uncertain or vague reasoning and has found applications in physical, control, biomedical, and linguistic sciences (23). In this approach, node interactions are described by continuous-valued logical propositions where the expression values of the variables, *q_i_*, can be any real number ranging from completely false, 0, to totally true, 1 (13, 14, 24). The corresponding fuzzy interaction rules may be straightforwardly derived from their Boolean counterparts by replacing ordinary logic connectors by algebraic expressions, as shown in **Figure 1**. The kind of approach has been previously employed by some of the present authors to describe differentiation and plasticity processes in floral organs (13), pancreatic beta cells (25), and CD4 T-cells (26–28).

To proceed, we denote the expression levels of the network agent *i* at a time *t* by a continuous variable *q_i_*(*t*), with 0 ≤ *q_i_* ≤ 1. The network dynamics is then described by a set of ordinary differential equations (ODEs):

(1) $$dq_{i}/dt=\mu [w_{i}(q_{i},\ldots q_{n})]-\alpha_{i}q_{i}.$$

Here, the input $\mu [w_{i}]=1/(1+\text{exp}[-\beta (w_{i}(q_{i},\ldots q_{n})-\theta_{th})])$ is a sigmoid function representing the truth value of the fuzzy interaction rule *w_i_* with respect to a threshold *θ_th_*, and *β* is a saturation rate. The second term yields an interaction decay at a rate $\alpha_{i}=1/\tau_{i}$, where *τ_i_* is a characteristic expression time. In this work, we assume *β* = 10, *θ_th_* = 1*/*2, and *α_i_* = 1. The differentiation dynamics induced by any considered cell microenvironment can be simulated by solving the ODE system (1) for a fixed set of initial conditions {*q_i_*(0)}. Solutions exhibit a long-time steady behavior, $q_{i}^{T}=q_{i}(t=T\gg 1)$, giving rise to a set of steady-state solutions $\left\{q_{i}^{T}\right\}$, as it will be shown in Results. In the simulations reported here, *T* = 30 is time long-enough long time long-enough. Alternatively, the set of steady-state solutions $\left\{q_{i}^{ss}\right\}$, may be derived from the condition *dq_i_/dt* = 0, yielding


$$q_{i}^{ss}=\frac{1}{\alpha_{i}}\;\mu [w_{i}(q_{1}^{ss},\ldots ,q_{n}^{ss})],$$

and we may identify $q_{i}^{ss}=q_{i}^{T}.$ Accordingly, the steady-state expression level of any network’s agent is determined by the truth value of its steady logical connections, modulated by its decay rate *α_i_*. In the case $\alpha_{i}\gg 1$, then $q_{i}^{ss}\approx 0$.

### 2.2 Simulation setup, data analysis and visualization

Simulations of the regulatory network dynamics were performed using a Python code involving numerical solvers for the ODEs described above; specifically, the ‘odeint‘ function from the ‘scipy‘ library. This allowed the implementation of an interactive program *Final monocyte network.ipynb* that may be used as a tool in the *Google Colab* application to test the dynamical expression of the network components under different microenvironmental and initial conditions. In particular, the initial conditions defining an unstimulated monocyte, {*q_k_*(0)}, were set to zero for most input variables (except for AMPK and GSK3β activities, which were set to 1). Then, different stimulation conditions were applied, including LPS to activate TLR4, ssRNA to activate TLR7, IgG immune complexes to engage Fc*γ* receptors, and combinations of these with cytokines (e.g. IL-4, IL-10). Each simulation was carried out over a predefined time interval, capturing the evolution of cell-produced cytokine levels, metabolic state, and transcription factor activities. The final steady states were analyzed to evaluate macrophage polarization markers under different stimulation conditions. Key variables, including cytokine expression, metabolic activity, and transcriptional factors dynamics, were plotted as time-series data to visualize the trajectories of monocyte activation and differentiation. Visualization was performed using the ‘matplotlib‘ library. Comparative analyses across conditions highlighted the influence of receptor activation and microenvironmental factors on the macrophage phenotype.

The results provided by the model have been validated against experimental data reported in the literature, ensuring consistency in cytokine profiles, metabolic shifts, and receptor-mediated differentiation. Sensitivity analyses were conducted to assess the robustness of the results to variations in parameter values. The Python code used for simulations is available upon request, ensuring reproducibility and transparency for future studies.

### 2.3 Robustness analysis

Robustness implies the capacity of a complex network to maintain its functionality subject to the action of topological or dynamical perturbations. Here, we assume that the network topology is already defined by selective forces, and we thus constrain our analysis to two different perturbation sources: i) the influence of randomly distributed initial values of the microenvironmental and cell variables on the differentiation stability. ii) the effect of noisy perturbations on the intrinsic differentiation dynamics, for fixed values of the initial microenvironment and cell variables.

#### 2.3.1 Effect of random distribution of initial values

To assess the stability of the system under parameter uncertainty we may consider a robustness index, *R*, that quantifies how much the model’s steady-state solutions change when several parameters are jointly perturbed (29). By assuming that the baseline values of the set of system parameters of interest are described by the vector $p=(p_{1},\ldots ,p_{M})$, then the steady state solutions of Equation 1 associated to a parameter set p and initial values $q_{0}$, can be denoted as $q_{i}^{ss}(p)=w_{i}(q^{ss};p)/\alpha_{i}$. In this context, we may evaluate the stability of our original predictions due to influence of random parameter perturbations, $p\to p+\delta p^{\left(k\right)}$, within a range $∥\delta p^{\left(k\right)}∥\leq \epsilon_{R}$, where *k* indicates the number of a given simulation. Then, for a given choice of $\epsilon_{R}$, the perturbed solutions are obtained from the ODE system:


$$dq_{i}/dt=\mu [w_{i}(q;p+\delta p^{\left(k\right)})]-\alpha_{i}q_{i},$$

with steady state solutions, $q_{i}^{ss}(\delta p^{\left(k\right)})=\mu [w_{i}(q^{ss};p+\delta p^{\left(k\right)})]/\alpha_{i}$. By making the identification $q_{i}^{ss}=q_{i}^{T}$, we may evaluate the deviation of the asymptotic perturbed solutions with respect to the original solution,


$$d_{k}=∥q^{T}(p+\delta p^{\left(k\right)})-q^{T}(p)∥/∥q^{T}(p)∥,$$

where $∥q∥=\sqrt{\sum_{i=1}^{n}q_{i}^{2}}$ denotes the usual Euclidean norm. Then, the robustness index is determined by the expression.


$$R=1-(1/N)\sum_{k=1}^{N}d_{k},$$

where *N* is the total number of realizations of the perturbation procedure, and 0 ≤ *R* ≤ 1.

The robustness index was evaluated for random variations of initial levels of phenotype-inducing microenvironments: M1 (LPS and IFNG-*γ*), M2a (IL-4), M2b (LPS and IL-1*β*) and M2c (IL-10), subject to three different perturbation levels, *ϵ_R_* = 0.1, 0.2, 0.3, with *T* = 30 time units and *N* = 500 iterations per considered scenario. Additionally, we performed a robustness test inducing perturbations only of the saturation response rate, *β*, an important parameter defining the rate at which the network elements can increase their activity.

#### 2.3.2 Effect of noise on differentiation efficiency

We evaluate the differentiation efficiency, $\epsilon_{D}$, defined as the fraction of cells reaching optimal expression levels (∼ 1) of phenotype-specific transcription factors (and associated cytokines) when the dynamic variables of the system are perturbed by stochastic interactions (28). This parameter may take values in the range $0\leq \epsilon_{D}\leq 1$, with $\epsilon_{D}=1$ corresponding to an idealized situation in absence of noise. For simplicity, in the following we the denote the expression level set {*q_i_*} by a vector **q** = (*q*_1_*, .., q_n_*). Noise perturbations are introduced in the description by introducing stochastic interactions *ξ_i_*(*t*) into the original ODE system (1):

(2) $$dq_{i}/dt=\mu [w_{i}(q)]-\alpha_{i}q_{i}+\xi_{i}(t).$$

Here, *ξ_i_*(*t*) is a random Gaussian variable, with null average ⟨*ξ_i_*(*t*)⟩ = 0, and a very short-time correlation ⟨*ξ_i_*(*t*)*ξ_i_*(*t' *)⟩=2*Qδ*(*t*–*t' *), where *Q* is a measure of the noise intensity, while *δ* is a Dirac-delta distribution centered at time $t=t^{\prime }$. Using this methodology, we analyzed the monocyte differentiation efficiency by introducing different levels of noise, *Q*, into the dynamical Equation 2 and performed 1,000 iterations. Average and standard deviation were calculated in order to estimate $\epsilon_{D}$ under each assayed condition.

### 2.4 Network’s modules

The network, displayed in **Figure 2**, includes nodes and green or red arrows that represent activating or inhibitory node interactions. The specific exogenous inputs are listed in **Table 1**, while the resulting Boolean interaction rules are presented in **Table 2**. For a visual assessment of the built-in elements, the network is presented as interconnected modules, whose different colors denote specific functions: extracellular stimuli, TLR’s, activating IgG immune complexes, cytokine receptors, downstream signals, metabolism, transcription factors, and secreted cytokines (output cytokines). Details of particular interactions can be visualized using the Yed software as *MONOCYTE NET.graphml* in the *https://grci.mx* web site.

**Table 1:** Extracellular stimuli (inputs).

Input	Notation	Description
Lipoprotein (TLR2 ligand)	LP	Exogenous lipoprotein stimulating TLR2 signaling.
Double-Stranded RNA	DSRNA	Viral dsRNA acting as a TLR3 agonist.
Single-Stranded RNA	SSRNA	Viral ssRNA acting as a TLR7/8 agonist.
CpG DNA	CPGDNA	Unmethylated CpG motifs acting as a TLR9 agonist.
Interferon Gamma	IFNGE	Cytokine input for IFN-*γ* receptor activation.
GM-CSF	GMCSFE	Cytokine that activates the CSF2 receptor (GM-CSF signaling).
Interleukin-1 Beta	IL1BE	Pro-inflammatory cytokine input.
Lipopolysaccharide Endotoxin	LPSE	LPS stimulus for TLR4 activation.
Interleukin-4	IL4E	Cytokine promoting M2 (regulatory) polarization.
Interleukin-10	IL10E	Anti-inflammatory cytokine input.
Inactivated C3b	IC3B	Complement fragment contributing to opsonization.
Immunoglobulin G	IGGC	IgG input for Fc*γ* receptor (FCGR)
Immunoglobulin A	IGAC	IgA immune complex influencing the activity of the Fc*α* receptor.
Oxygen	O2	Extracellular oxygen concentration.
Fatty Acids	FA	Availability of fatty acids for metabolism.
Glucose	GLC	Glucose concentration in the extracellular environment.
Cytosolic Double-Stranded RNA	CITDSRNA	Activator of the RIG-I pathway (dsRNA signal).
Cytosolic Single-Stranded RNA	CITSSRNA	Activator of the RIG-I pathway (ssRNA signal).
Tumor Necrosis Factor *α*	TNFA	Pro-inflammatory cytokine input.
Cellular Inhibitor of Apoptosis	CIAP	Regulator modulating TNF receptor downstream signaling

**Table 2:** Boolean rules*, **.

Standard nomenclature	Node name	Boolean rule	References
IFN-*γ*R	IFNGR	IFNGE	10.1038/s41577-018-0029-z
GM-CSFR*α*	CSF2RA	GMCSFE	10.3109/08977194.2011.649919
IL-1R	IL1R	IL1BE | IL1BOUT	10.1146/annurev.immunol.021908.132612
TLR4	TLR4	LPSE	10.1016/j.preghy.2020.06.002
Fc*γ*R	FCGR	IGGC | (IGGC & IL1BE) | MINCLE	10.3389/fimmu.2020.01393; 10.3389/fimmu.2017.00280; 10.1111/imm.12167; 10.1016/j.immuni.2013.03.010
IL-4R*α*	IL4RA	IL4E	10.1046/j.1365-2567.1999.00711.x
IL-10R	IL10R	IL10E | IL10OUT	10.3389/fimmu.2023.1188750
STAT1	STAT1	IFNGR &!(SOCS1 | STAT3)	10.1038/s41392-021-00791-1; 10.1038/s41392-023-01468-7; 10.2147/CMAR.S182105
STAT5	STAT5	CSF2RA &!(STAT3 | IRF4 | SOCS1)	10.3389/fphys.2018.01659; 10.1038/s41392-021-00791-1
NF- κB	NFKB	IL1R | NEMOIKKB | CARD9 | AKT | PKC | (RIP1 & TRAF2 & CIAP) &!(STAT3 | PPARG | KLF4 | CREB1)	10.3389/fphys.2018.01659; 10.1182/blood-2010-07-273417; 10.1146/annurev.immunol.16.1.225; 10.1073/pnas.97.7.3394; 10.1038/nature04926; 10.1038/43466; 10.3892/ijo.2014.2578; 10.1126/science.1071924
PPAR*γ*	PPARG	IL4RA	10.1038/nature05894
STAT6	STAT6	IL4RA &!STAT1	10.3389/fphys.2018.01659; 10.4049/jimmunol.164.5.2303
JMJD3	JMJD3	IL4RA	10.1038/ni.1920S
STAT3	STAT3	(IL10R | (ERK & P38)) &! (PPARG | SOCS3)	10.1242/dmm.024745; 10.1146/annurev.immunol.19.1.683; 10.4049/jimmunol.175.1.469
SOCS3	SOCS3	NFKB | STAT1	10.1038/s41392-023-01452-1
IRF3	IRF3	TBK1IKKI | NEMOTBK1IKKE	10.1038/ni921; 10.1074/jbc.M205069200
ERK	ERK	FCGR | MKK1/2 | PI3K	10.1038/nri2206
KLF4	KLF4	STAT6	10.1172/JCI45444
SOCS1	SOCS1	STAT6 | IFNABOUT	10.1074/jbc.M403223200
IRF4	IRF4	JMJD3	10.1038/ni.1920
Produced IL-1*β*	IL1BOUT	NFKB | (NFKB & AP1)	10.1038/sigtrans.2017.23
Produced IL-12	IL12OUT	STAT1 | (STAT5 & NFKB) | (AP1 & NFKB)	10.1038/nri1001
TAK1/2/3	TAK123	MYD88	10.14348/molcells.2023.2193
JNK/MAPK	JNKMAPK	TAK123	10.14348/molcells.2023.2193
ERK1/2	ERK12	JNKMAPK	10.14348/molcells.2023.2193
MSK1/2	MSK12	ERK12 | P38	10.14348/molcells.2023.2193
GSK-3*β*	GSK3*β*	!AKT3 |!AKT1	10.14348/molcells.2023.2193; 10.1186/s12950-023-00360-z; 10.4049/jimmunol.1601515
Produced IL-10	IL10OUT	PPARG | STAT6 | JMJD3 | STAT3 | (CREB1 & AP1)	10.1038/nri2711; 10.14348/molcells.2023.2193; 10.1016/j.smim.2019.101324; 10.7554/eLife.85964; 10.1016/j.cmet.2007.06.010; 10.1016/j.intimp.2020.107266
Akt1	AKT1	PI3k &!IFNGR	10.1186/s12950-023-00360-z
Akt3	AKT3	PI3k &!IFNGR	10.1186/s12950-023-00360-z
TLR2	TLR2	LP	10.1189/jlb.0907656; 10.1016/j.immuni.2009.11.008
TLR3	TLR3	DSRNA	10.1089/jir.2014.0034
TLR7	TLR7	SSRNA	10.1073/pnas.0400937101
TLR8	TLR8	SSRNA	10.1016/j.bmc.2017.11.020
TLR9	TLR9	CPGDNA	10.2174/1566524023362159
MyD88	MYD88	TLR2 | TLR4 | TLR7 | TLR8 | TLR9	10.1038/ni.1863; 10.1038/35100529
IRAK4	IRAK4	MYD88	10.1016/j.immuni.2008.09.015
IRAK1/2	IRAK1/2	IRAK4	10.1074/jbc.M700548200; 10.1084/jem.20061523
TRAF6	TRAF6	IRAK1/2	10.3389/fimmu.2023.1133354
TAB2/3/TAK	TAB2/3TAK	TRAF6 | RIP1	10.3389/fimmu.2020.608976; 10.1038/nature08247
MKK3/6	MKK3/6	TAB2/3TAK	10.1152/ajpheart.00186.2005; 10.15252/embr.201948035
p38	P38	MKK3/6	10.1016/j.bbamcr.2007.03.010
CREB1	CREB1	MSK1/2 &!GSK3*β*	10.14348/molcells.2023.2193
MKK4/7	MKK4/7	TAB2/3TAK	10.1016/j.tips.2012.06.007
JNK	JNK	MKK4/7 | PI3K	10.4049/jimmunol.180.6.4218; 10.1007/s00018-013-1322-4; 10.4049/jimmunol.0801352
c-Jun	CJUN	JNK	10.1016/s0092-8674(00)00116-1
MKK1/2	MKK1/2	NEMOIKKB	10.1016/j.jbc.2022.101864
c-Fos	CFOS	ERK	10.1074/jbc.C500353200
AP-1	AP1	CJUN | CFOS | MSK12 &!GSK3*β*	10.1016/j.bbalip.2014.05.007; 10.1006/excr.2001.5180; 10.1038/nri2711
NEMO/IKK*β*	NEMOIKKB	TAB2/3TAK	10.1016/j.molcel.2004.08.008
RIP1	RIP1	TRIF | TRADD	10.1074/jbc.M506831200; 10.1016/j.bbrc.2013.12.068
Endosomal TLR4	TLR4END	TLR4	10.2337/db21-0426
TRIF	TRIF	TLR3 | TLR4END	10.1631/jzus.B2000808; 10.1074/jbc.M506831200
TRAF3	TRAF3	TRIF | (TRADD & MAVS) | (IRAK1/2 & TLR7) | (IRAK1/2 & TLR8) | (IRAK1/2 & TLR9)	10.3389/fimmu.2019.00104; 10.1111/j.1600-065X.2011.01055.x; 10.1038/cr.2011.2; 10.3389/fimmu.2014.00461
TBK1/IKK ∈	TBK1IKKI	TRAF3 & TRIF	10.1038/nri2998; 10.1074/jbc.M311629200
IKK*α*	IKKA	TRAF3 & IRAK1/2	10.1074/jbc.M109.076091; 10.3389/fimmu.2014.00553
IRF7	IRF7	IKKA | NEMOTBK1IKKE	10.1016/j.molimm.2007.10.034; 10.1038/ni1465
Produced IL-6	IL6OUT	NFKB | (AP1)	10.1093/cvr/cvq076; 10.1097/00024382-200014030-00025; 10.3389/fimmu.2016.00604
Produced IL-18	IL18OUT	NFKB & AP1	10.4049/jimmunol.1001829; 10.1016/j.cyto.2014.05.003; 10.1016/S0006-291X(02)02433-6
Produced IL-33	IL33OUT	NFKB & AP1	10.1038/ni.3772; 10.1002/eji.201040718
Produced IFN-*α*	IFNABOUT	IRF3 | IRF7	10.1016/s1074-7613(00)00053-4; 10.1016/j.molimm.2007.10.034
Phagocytosis	PHAGOCYTOSIS	(WAVE & WASP) | RAC	10.1155/2023/3577334; 10.1016/j.celrep.2016.09.039; 10.4161/cib.3.2.10759; 10.4161/sgtp.27952
Phagosome	PHAGOSOME	PHAGOCYTOSIS	10.1097/MOT.0000000000000313
Processing	PROCESSING	PHAGOCYTOSOME | ENDOSOME	10.1016/j.coi.2007.10.010; 10.1146/annurev-immunol-032712-095910
MHC-II	MHC2	PROCESSING	10.1146/annurev-immunol-032712-095910
ITAM	ITAM	FCGR	10.1146/annurev.immunol.15.1.203
Syk	SYK	ITAM | SRC | CA | DAP12 | (HCK & FGR)	10.1073/pnas.94.5.1919; 10.1074/jbc.M804942200; 10.1016/j.molcel.2008.06.023; 10.1182/bloodadvances.2021006147
CARD9	CARD9	SYK	10.1016/j.immuni.2018.08.024
Vav	VAV	SYK	10.1016/s1074-7613(00)80273-3
Rac	RAC	VAV	10.1128/MCB.25.10.4211-4220.2005; 10.3389/fimmu.2019.02585
Cdc42	CDC42	VAV	10.1161/ATVBAHA.118.312087
WASP	WASP	RAC	10.1016/j.cub.2019.10.036
WAVE	WAVE	CDC42	10.3389/fmicb.2018.00360
Ca2+	CA	PLC	10.1038/sigtrans.2017.23; 10.1016/j.bbamcr.2021.119040
Lyn	LYN	FCAR	10.1016/j.exppara.2020.107970; 10.1111/j.1600-065X.2008.00758.x
Fyn	FYN	FCAR	10.1111/j.1600-065X.2008.00758.x
DAP12	DAP12	FYN | LYN	10.1016/j.cellsig.2012.02.014; 10.1002/alz.088509
PI3K	PI3K	SYK | MYD88	10.1074/jbc.M111.255125; 10.14348/molcells.2023.2193
PLC	PLC	SYK	10.1016/s1074-7613(00)80012-6
Akt	AKT	PI3K	10.3892/mmr.2018.9713
PKC	PKC	PLC	10.1182/blood.V72.2.739.739
CD11b/CD18	CD11BCD18	IC3B	10.1073/pnas.91.22.10680
Hck	HCK	CD11BCD18	10.1016/j.jare.2023.02.010; 10.1016/j.febslet.2006.06.099
Fgr	FGR	CD11BCD18	10.1038/s41420-023-01538-3
Fc*α*R	FCAR	IGGA	10.1517/14728222.2014.877891
mTOR	MTOR	AKT | NFKB	10.1007/s12282-024-01567-5; 10.1038/sigtrans.2017.23
mTORC1	MTORC1	(MTOR & (AKT | NFKB) &!AMPK)	10.1242/jcs.051011
mTORC2	MTORC2	(MTOR & AMPK) | (MTOR & IL4E)	10.1126/scisignal.aav3249; 10.1126/scisignal.267pe27; 10.1016/j.immuni.2016.09.016
LKB1	LKB1	(AKT & AMPATPratio) | IL4E | IL10E	10.3389/fmicb.2019.00520; 10.4049/jimmunol.181.12.8633
AMPK	AMPK	((LKB1 | (CA | AKT) & AMPATPratio) &!MTORC1)	10.3389/fmicb.2019.00520; 10.4049/jimmunol.181.12.8633
Glycolysis	Glycolysis	((MTORC1 | HIF1A) & GLC) &!AMPATPratio	10.3892/ijo.2020.5152; 10.1113/JP280572
OXPHOS	OXPHOS	AMPK & FA	10.1038/nrm.2017.95; 10.1074/jbc.M110.139493
AMP/ATP ratio	AMPATPratio	Glycolysis &!OXPHOS	10.3945/ajcn.110.001925
HIF-1*α*	HIF1A	!O2 & AKT	10.1016/j.micinf.2016.11.003; 10.1038/s41523-023-00598-z
RIG-I	RIG1	CITDSRNA | CITSSRNA	10.1084/jem.20081210; 10.1016/j.molcel.2022.11.018
MAVS	MAVS	RIG1	10.1016/j.coviro.2015.04.004
TRADD	TRADD	MAVS | TNFR1	10.1371/journal.ppat.1004020; 10.1016/0092-8674(95)90070-5
NEMO/IKK*α*/*β*	NEMOIKKAB	RIP1 & TRADD	10.1016/j.molcel.2006.03.026
NEMO/TBK1/IKK ∈	NEMOTBK1IKKE	TRAF3 & TRADD	10.1016/j.immuni.2008.03.013
TNFR1	TNFR1	TNFA | TNFAOUT	10.1615/critreveukargeneexpr.v20.i2.10
TRAF2	TRAF2	TRADD	10.1016/s0092-8674(00)80984-8
Produced TNF*α*	TNFAOUT	NFKB	10.1016/j.smim.2014.05.004
Dectin-1	DECTIN1	NFKB	10.18632/aging.20492; 10.1038/ni.1692
Dectin-2	DECTIN2	NFKB	10.1101/cshperspect.a002352
Mannose receptor	MR	NFKB	10.3389/fimmu.2021.765034
CLEC10A	CLEC10A	NFKB	10.1038/nri.2016.55
Mincle	MINCLE	NFKB	10.3389/fimmu.2017.00861; 10.1158/2326-6066.CIR-19-0782

*Logical connectives symbols: and → &, or → |, not →!. ** Citations for DOI’s are provided in **Supplementary Table 1**.

Inflammatory pathways are represented in **Figure 3A**, which displays the convergence of activating and inhibitory signals on the NF*κ*b node, as well as the consequent production of inflammatory cytokines (TNF*α*, IL-1*β*, IL-12, IL-6, IL-18, and IL-33). This module includes the activity of metabolic elements such as mTOR and mTORC1, as well as transcription factors and cofactors such as CREB1, AP-1, IRF3 and IRF7 that are either activated by the TNF*α* and FC*γ* receptors, or by TLR engagement. Since the effects of Akt on macrophage polarization appear to be Akt isoform-specific (30), its isoforms Akt1, Akt2 and Akt3, have been incorporated in the network regulatory pathways (**Figure 3B**). In particular, experiments with macrophages deficient in Akt1 or Akt2 revealed that this default gives rise to M1 and M2 macrophages, respectively (31).

The pathways leading to the anti-inflammatory role of CREB1 are shown in **Figure 3B**, highlighting GSK3*β* as inhibitor of the CREB1 function. It can be observed that this module displays the downstream pathway:


IFNGE → IFNGR ↛(AKT1 or AKT3)↛ GS3KB ↛CREB1 ↛NFKB

which involves four consecutive inhibitory interactions, leading to the activation of the last downstream element; therefore, the presence of IFN-*γ* in the cell microenvironment promotes the activation of NF-*κ*B. Conversely, in the absence of IFN-*γ*, an anti-inflammatory macrophage profile may arise, as may be inferred from the middle factors in the route: upon activation (by PI3K) the Akt1 and Akt3 mediators hinder the action of the constitutively expressed factor GSK3*β* (32), leading to anti-inflammatory effects of CREB1 through NF-*κ*B inhibition.

**Figure 3C** shows a module for AMPK-controlled glycolysis and oxidative phosphorylation (OXPHOS), reflecting the metabolic profiles of M1 and M2 phenotypes. In this module, AMPK is a central energy sensor of the AMP/ATP ratio and involves a negative feedback loop with mTORC1, defining a switch driving either OXPHOS or glycolytic activity.

The signaling pathways induced by the exogenous immunoregulatory cytokines IL-4 and IL-10 are shown in **Figure 4**. Receptor signaling by these cytokines leads to inhibition of NF*κ*b, promotes the production of IL-10 and the activation of AMPK, directing the macrophage metabolism toward OXPHOS.

## 3 Results

### 3.1 *In silico* reproduction of canonical macrophage phenotypes

**Figure 5:**
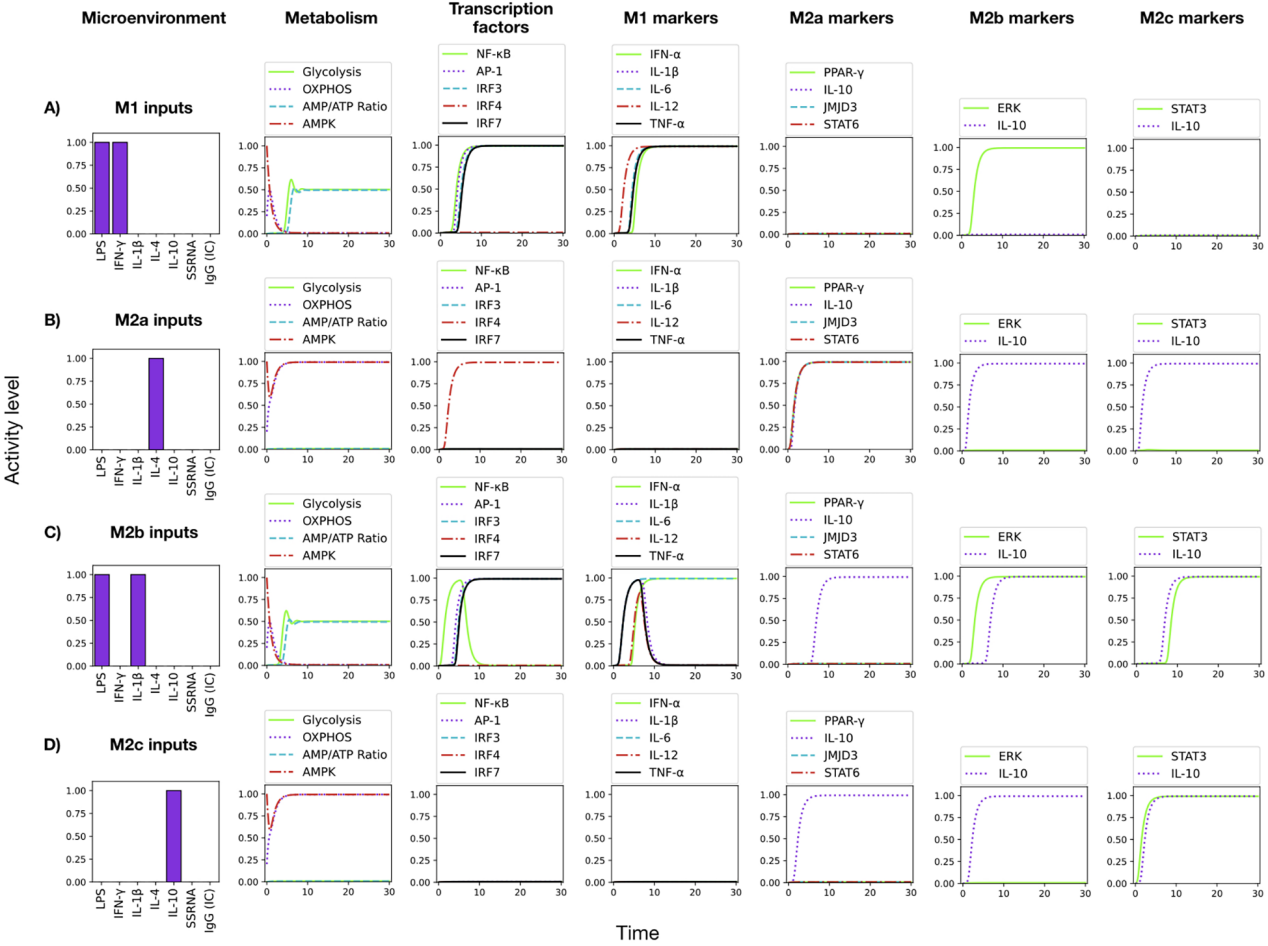
Dynamical evolution of monocyte differentiation into the M1, M2a, M2b, and M2c phenotypes induced by microenvironment stimuli. **(A)** LPS+IFN*γ* combination induces full M1 differentiation with a glycolytic metabolism and induction of pro-inflammatory cytokines. **(B)** IL-4 stimulation alone drives M2a expression markers with OXPHOS activity and IL-10 expression. **(C)** LPS+IL-1*β* combination induces a mixed M2b/M2c phenotype, with a glycolytic metabolism, transient production of pro-inflammatory cytokines and steady expression of IL10. **(D)** IL-10 stimulation induces an earlier expression of M2c markers, with an oxidative metabolism and further IL-10 production.

**Figure 6:**
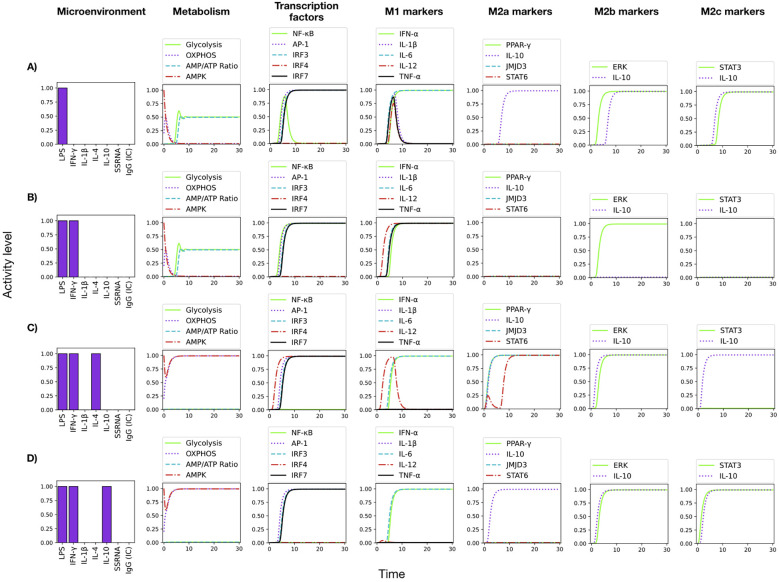
Predicted effects of IL-4 and IL-10 on M1 differentiation. **(A)** LPS induces a mixed M1/M2b/M2c phenotype, with production of pro- and anti-inflammatory cytokines. **(B)** LPS + IFN-g drives a strong M1 polarization, with a glycolytic metabolism and production of all pro-inflammatory cytokines. The M2a and M2c phenotypes are inhibited, while M2b is partially suppressed. **(C)** IL-4 abrogates the activity of NF-γB, with a shift to a mixed M2a/M2b phenotype, with a OXPHOS metabolism and induction of both pro- and anti-inflammatory cytokines. **(D)** IL-10 abrogates the activity of NF-γB and IRF4, with a shift to a mixed M2b/M2c phenotype, with an OXPHOS metabolism and induction of both pro- and anti-inflammatory cytokines.

**Figure 7:**
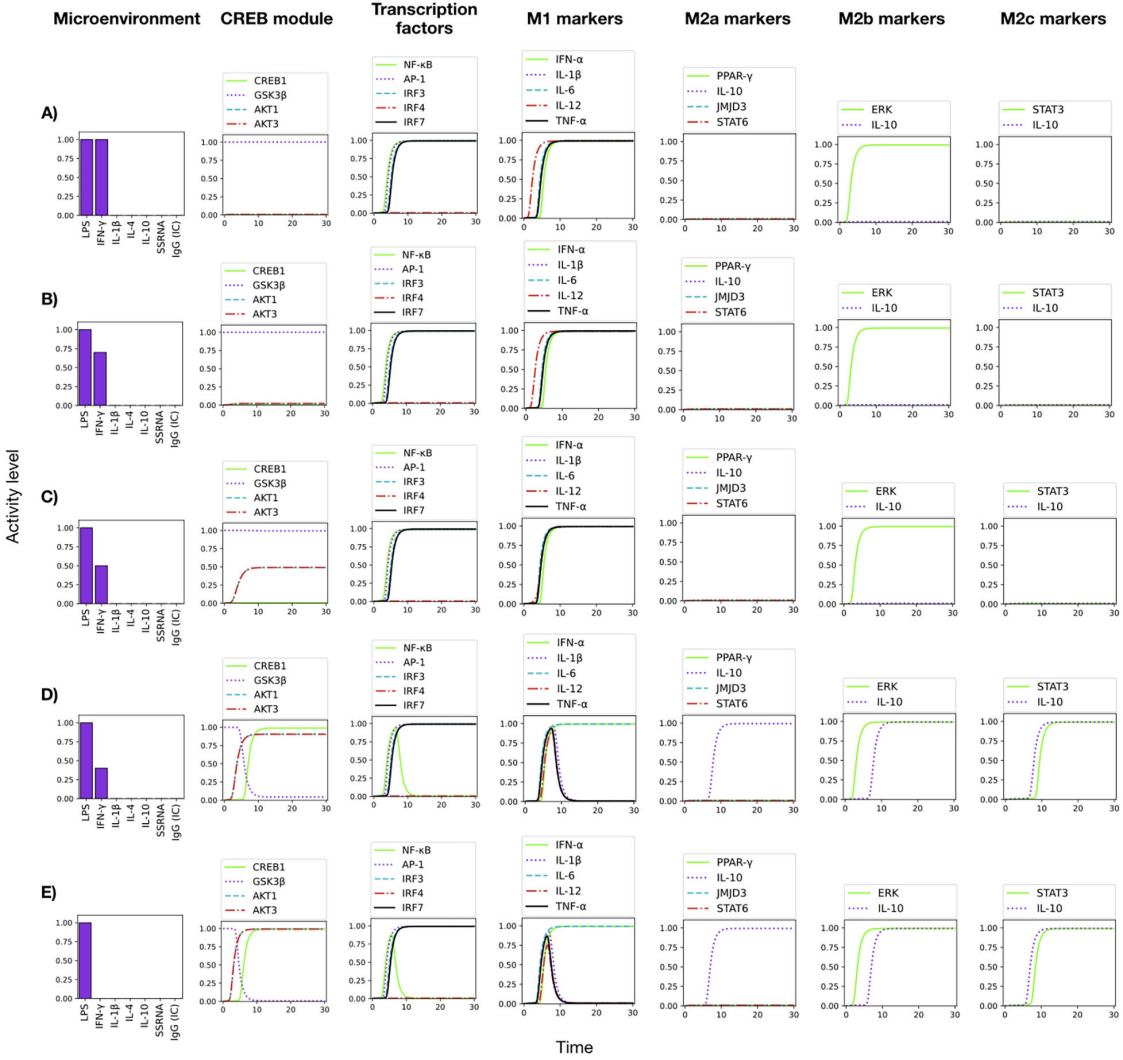
Effect of variable levels of IFN-*γ* on CREB1 and NF*κ*B in the pro- and anti-inflammatory macrophage differentation. **(A)** The combination of exogenous LPS and IFN-*γ* at optimal levels (LPS = 1, IFN-*γ* = 1) leads to a prevailing M1 polarization with induction of pro-inflammatory citokines. IFN-*γ* impedes de inactivation of the CREB1 inhibitor, GSK3*β*, allowing a stable NF-*κ*B activity. **(B)** LPS + 0.75 IFN-*γ* still yields an identical expression pattern as in the previous case. **(C)** LPS + 0.50 IFN-*γ*, also leads to a pro-inflammatory pattern, but now AKT1 and AKT3 are expressed at a middle level. **(D)** LPS + 0.25 IFN-*γ*, induces the expression of AKT1 and AKT3 at optimal levels, so that GSK3*β* is depleted, allowing the expression of CREB1 and the concomitant inhibition of NF-*κ*B. So, the M1 polarization is disrupted, and a mixed M2b/M2c phenotype is generated. **(E)** LPS alone yields identical results as **(D)**.

**Figure 8:**
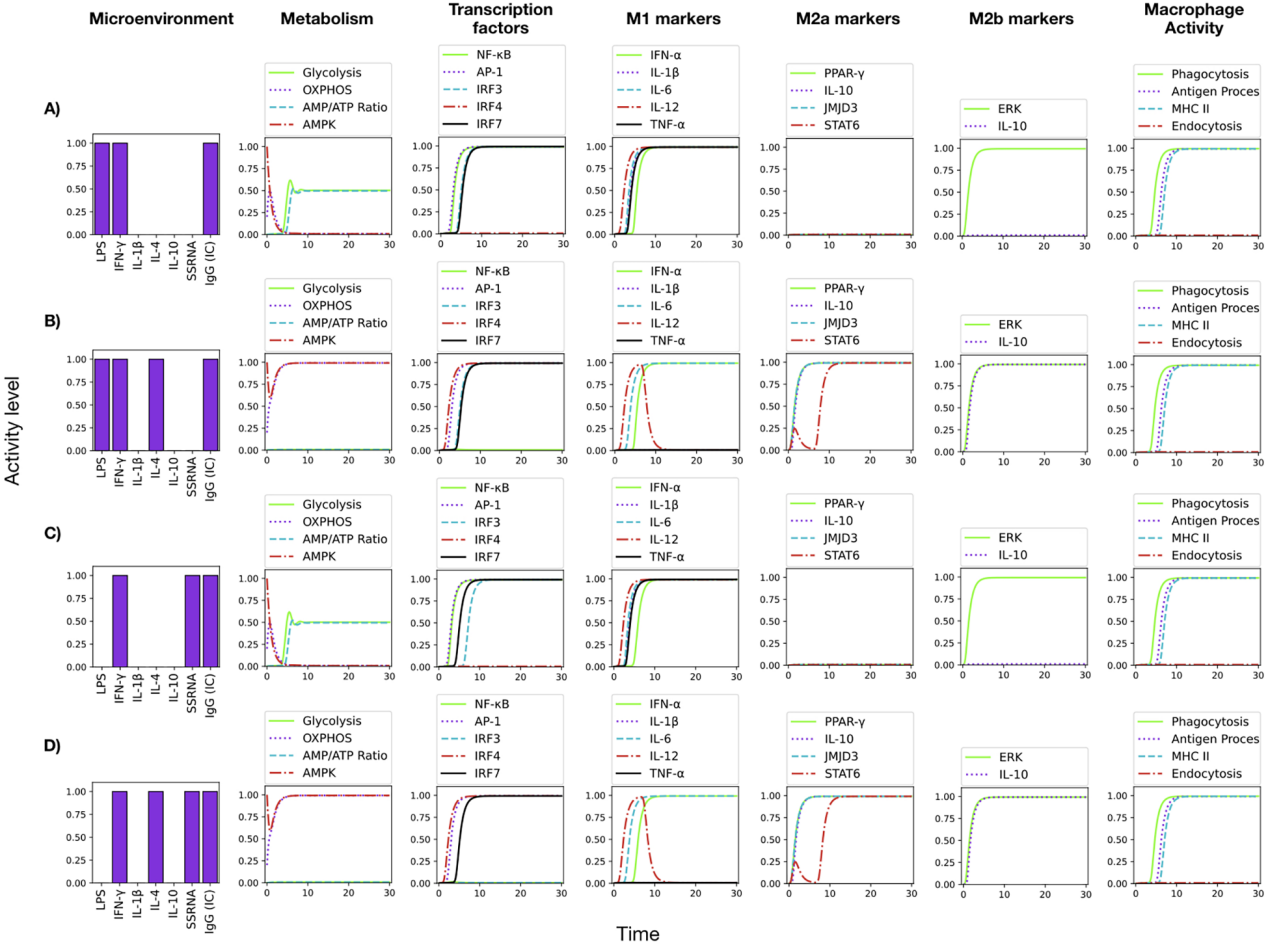
Induction of phagocytosis and antigen presentation by IgG immune complexes (IC). Addition of IC induces phagocytosis and antigen processing and presentation to the **(A)** M1 stimulus, **(B)** M1 stimulus+IL-4. **(C)** M1 stimulus composed by ss-RNA+IFN-γ, and **(D)** ss-RNA+IFN-γ stimulus in the presence of IL-4.

**Figure 9:**
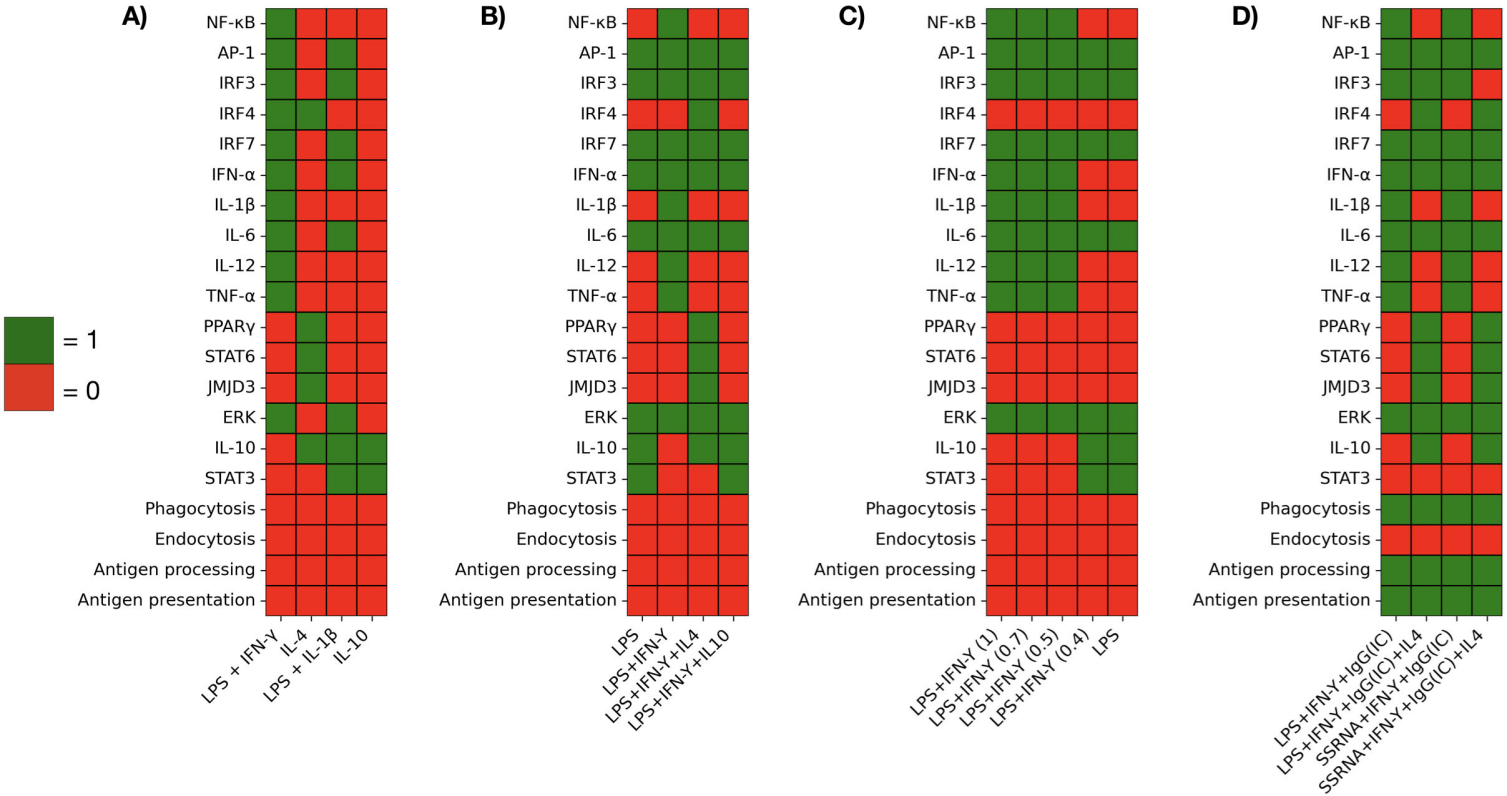
Summary of steady-state values of nodes (asymptotic states at a time T = 30) in the microenvironments analyzed in **Figures 5**–**8**. Columns show different treatment conditions. **(A)** M1, M2a, M2b, and M2c profiles induced by stimulation with LPS+INFgamma, IL-4, LPS+IL-1beta, and IL10, respectively. **(B)** Anti-inflammatory effect of IL-4 and IL-10 on the M1profile. **(C)** Effect of variable levels of INFgamma on the M1 profile. **(D)** M1 profiles obtained by stimulation with LPS+INFgamma and ssRNA+INFgamma. Effect of IL-4 and induction of phagocytosis and antigen presentation by IgG immune complexes (IC).

We applied the mathematical model to the analysis of a set of relevant initial conditions introduced as inputs of the system (see **Table 1**). **Figures 5**-**8** depict the time evolution of network components induced by diverse cell microenvironments, and **Figure 9** summarizes the stable states obtained.

The network faithfully reproduces the spectrum of macrophage functional states, known as M1, M2a, M2b, and M2c. **Figure 5** displays the dynamics of metabolic changes, transcription factor activity, cytokine production and markers associated with the M1, M2a, M2b and M2c phenotypes. It can be observed that co-stimulation with LPS and IFN-*γ* drives a full M1 polarization (**Figure 5A**). The system converges to a sustained activation of NF-*κ*B, a marked increase in glycolytic flux, and robust production of all pro-inflammatory cytokines (IFN-*α*, IL-1*β*, IL-6, IL-12 and TNF-*α*). This outcome reproduces the metabolic and secretory profile typical of classically activated macrophages (3, 33). On the other hand, IL-4 alone (**Figure 5B**) promotes a M2a state: AMPK-driven oxidative phosphorylation prevalence over glycolysis, activity of PPAR-*γ*, JMJD3, and STAT6, and production of IL-10. These features mirror the anti-inflammatory and tissue-repair phenotype described for IL-4–stimulated macrophages (34, 35). When LPS is paired with IL-1*β* (**Figure 5C**), the model yields the M2b response: a transient burst of TNFα is induced (similarly to the case of LPS alone (**Figure 6A**) Gycolysis remains dominant, and a mixed set of pro- and anti-inflammatory cytokines is produced, with activation of Erk and STAT3 and production of IL-10. This phenotype recapitulates observations that LPS+ IL-1*β* stimuli can generate hybrid profiles (36, 37). Finally, IL-10 alone (**Figure 5D**) drives a full M2c program: sustained OXPHOS metabolism, strong GSK3*β* activation (not shown), and production of IL-10 through the activity of STAT3; pro-inflammatory cytokines are fully suppressed. This result aligns with the immunoregulatory profile induced by IL-10 *in vitro* and *in vivo*, and are consistent with tissue repair functions (3, 36, 37). Thus, the monocyte model reproduces the hallmark metabolic and cytokine signatures of canonical macrophage phenotypes.

### 3.2 Immunomodulation by IL-4 and IL-10

Signaling events related to immunomodulation by IL-4 and IL-10 were incorporated in the network, as seen in **Figure 4** and **Table 2**. IL-4 receptor signals for stimulation of the histone demethylase JMJD3, STAT6, and PPAR-γ, a known inhibitor of NF-κB. STAT6 activation leads to the expression of the IL-10 gene (34, 38–40). Likewise, STAT6 activates the Krüppel-like factor 4 (KLF4), which contributes to the inhibition of NF-*κ*B and therefore of the TNF-*α* synthesis. Thus, IL-4 can inhibit NF-*κ*B via PPAR-*γ* and KLF4 even in the presence of IFN-*γ*.

The addition of IL-4 to the LPS+IFN-*γ* stimulus promotes partial differentiation to the anti-inflammatory phenotypes M2a and M2b, due to the positive action of JMJD3 on the IRF-4 transcription factor and inhibition of STAT5 (**Figure 6C**). On the other hand, activation of STAT6 by IL-4 activates SOCS-1, an inhibitor of STAT1. In the model, inhibition of STAT5 and STAT1 reduces IL-12 production (**Figures 6C**), as reported (41, 42). M2 macrophages tend to depend more on mitochondrial oxidative phosphorylation and fatty acid oxidation than glycolysis (8, 35, 36, 43, 44). The preferential use of OXPHOS over glycolysis by IL-4 or IL-10-stimulated M2 macrophages is reproduced by the model (**Figures 6C, D**). Thus, the model reproduced de role of IL-4 and IL-10 in steering differentiation away from a strictly pro-inflammatory state.

### 3.3 IFN-*γ* drives TLR4-induced inflammation by promoting the activity of the CREB1-inhibitor GSK3*β*

The CREB1 inhibitor GSK3*β* is constitutively expressed in monocytes (19). Upon TLR4 stimulation, GSK3*β* is inhibited via the PI3k-Akt-dependent pathway, specifically through the Akt1 and Akt3 kinases, so that CREB1 remains active, inhibiting the activity of NF-*κ*B and promoting the production of IL-10 (**Figure 3B**) (reviewed by (45). The constitutive expression of GSK3*β* was incorporated in the model by asignyinig it an initial 1 value. Simulations showed that TLR4 activation by LPS alone drives the activity of Akt1 and Akt3, inhibition of GSK3*β* and induction of CREB1 activity (**Figure 7**). As a result, an incomplete pro-inflammatory profile is obtained, marked by a transient activation of NF-*κ*B, with a decay coincident with the induction of CREB1 activity. Accordingly, IL-1*β* and TNF-*α* are transiently produced. The production of IFN-*α* and IL-6, together with the activity of the transcription factors AP-1 and IRF7 and a glycolytic metabolism are observed.

The effect of variable levels of INF-*γ* on the M2b/M2c phenotype induced by LPS is shown in **Figures 7A-D**. INF-*γ* inhibits the protein kinases Akt1 and Akt3 activities, allowing the function of GSK3*β*, leading to CREB1 inhibition. In this condition, NF-*κ*B is stably active and a complete M1 cytokine profile is induced, as shown in (**Figure 7A**). Thus, modeling results are compatible with a relevant role of CREB1 in the inhibition of the pro-inflammatory response via inhibition of NF-*κ*B and promotion of IL-10 synthesis. The model predicts that a full activity of NF-*κ*B is maintained even when the levels of INF-gamma decreases to half of the optimum value (**Figures 7B-C**). However, lower amounts of this cytokine (0.25) allow the activity of AKT1 and AKT3, with the concomitant depletion of GSK3*β*, which in turn allows the activity of CREB1 and the subsequent inhibition of NF-*κ*B. As a result, the M1 polarization is disrupted, and mixed M2 phenotypes are generated. The absence of IFN-*γ* yields the same result (**Figures 7D, E**). Thus, the model shows that the stabilization of NF-*κ*B activity by IFN-*γ* can be reached through inhibition of the pathways leading to activation of the CREB1 inhibitor GSK3*β*, and highlights that GSK3*β* is necessary for the TLR4-mediated M1 inflammatory response, as suggested before Xia et al. (32)Ko and Lee (46).

### 3.4 Induction of phagocytosis by IgG-immune complexes and IL-4 effect

The binding of IgG-antigen immune complexes (IC) to Fc receptors induces strong phagocytosis, antigen presentation capabilities and production of pro-inflammatory cytokines in monocytes (47–49). **Figure 8A** shows the result of modelling the addition of IgG-antigen immune complexes to LPS+IFN-*γ*-activated monocytes. Besides the induction of all the pro-inflammatory cytokines, signaling through the Fc*γ* receptor induces the promotion of phagocytosis, antigen processing, and MHC class II presentation through the Syk-VAV-Rac pathway (47–49). As shown above, the presence of IFN-*γ* keeps CREB1 inactive and so NF-*κ*B is active.

**Figure 8B** shows that the input of IL-4 to the LPS+IFN-*γ*+IgG (IC) condition, blocks the NF-*κ*B function and TNF-*α* synthesis, while the activation of JMJD3 promotes the function of the IRF4 transcription factor. IRF4 inhibits STAT5, thus inhibiting IL-12 secretion; however, a transient production of this cytokine is still supported by STAT1, NF-*κ*B, and AP-1 activities (**Figure 8B**). IL-1 is not produced, as described (50). The production of IL-10 is induced by IL-4 respect to the previous condition and the metabolism is shifted to oxidative phosphorylation. As a whole, IL-4 leads to an M2 profile. The results of the modelling agree with the notion that IL-4 can counteract the stimulation by LPS, IgG-immune complexes, and IFN-*γ* in monocytes (51–53), whereas the phagocytosis and antigen processing capabilities are maintained.

Intracellular TLR7 and TLR8 recognize ssRNA and signal though the TRAF3 and MyD88 elements. Whereas signals downstream MyD88 induce the production of pro-inflammatory cytokines, TRAF3 induces the activity of interferon regulatory factors 3 and 7 (IRF3 and IRF7), which promote the production of type I interferons (IFN*α* and IFN*β*), essential components of the anti-viral response (54). These rules were included in the model (**Table 2**) and the effect of ssRNA stimulation was analyzed. Results showed that ssRNA-only stimulation induces a glycolytic metabolism and only a transient expression of NF-*κ*B and IRF-3 (see the interactive program *Final monocyte network.ipynb* in the *Google Colab* application). IFN-*γ* greatly potentiate the M1 pro-inflammatory profile (55–57), with a stable induction of NF-*κ*B and IRF3. IgG (IC)) added phagocytosis and antigen presentation activities to the ssRNA+IFN-*γ* situation (**Figure 8C**). The expression of M2 markers was not obtained. Therefore, the model integrates the view that IFN-*γ* supports the production of pro-inflammatory cytokines, whereas IgG immune complexes induce phagocytosis and antigen presentation capabilities during the ssRNA+IFN-*γ* response. As in the case shown in **Figure 8B**, the addition of IL-4 produces the inhibition of NF-*κ*B, a transient production of IL-12, and the expression of M2 markers. In addition, the expression of IRF3 is inhibited (**Figure 8D**).

### 3.5 Robustness of the macrophage differentiation process

**Figure 10:**
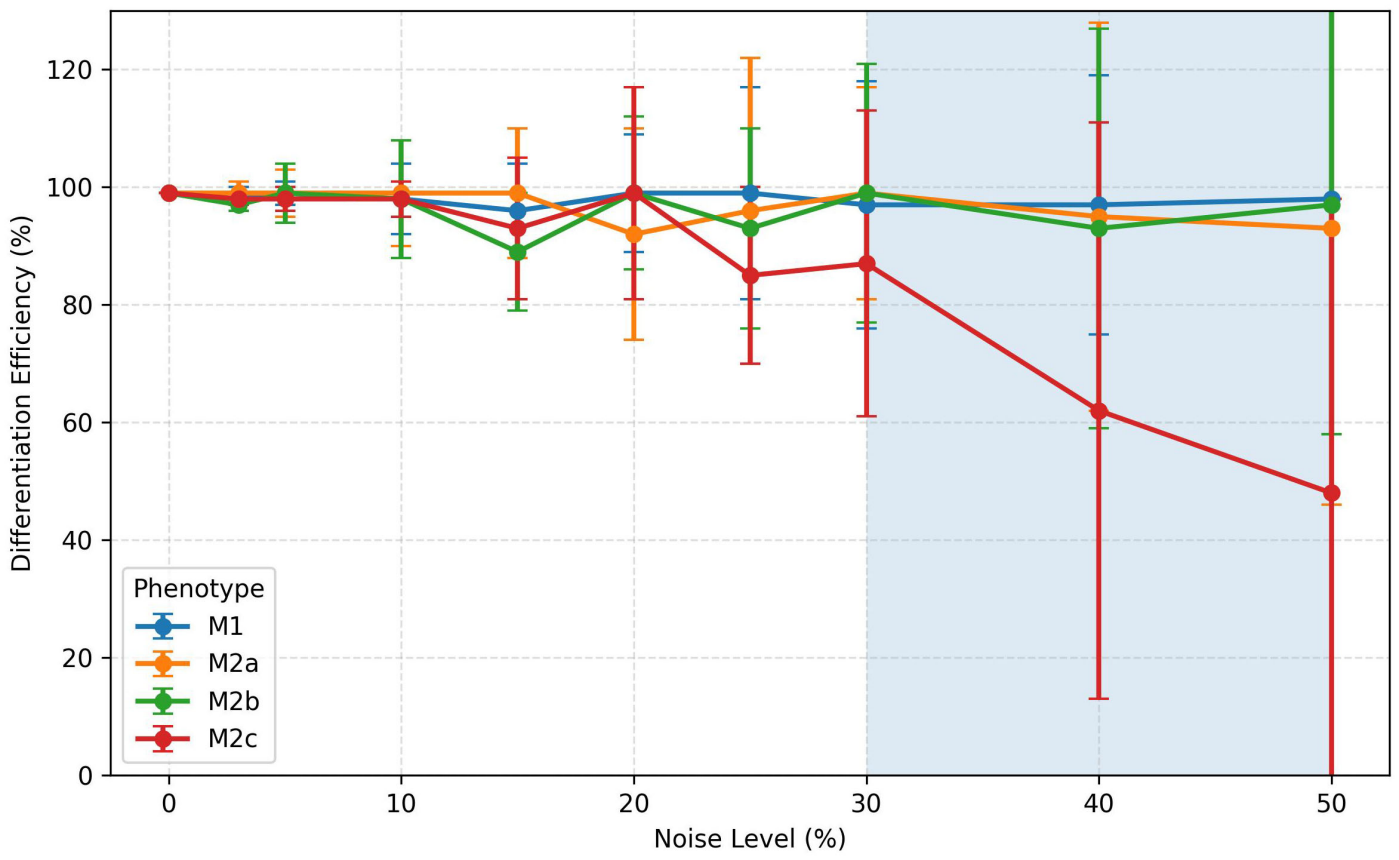
Effect of randomly distributed values of microenvironmental and cell nodes (stochastic noise) on macrophage differentiation efficiency under M1, M2a, M2b, and M2c input conditions. was calculated after 1,000 iterations. M1, M2a, and M2b remain stable around 100%. M2c declines significantly after 30% noise level. Error bars indicate standard deviation.

Stochastic models have been instrumental in exploring system stability in the face of fluctuations arising from random variations of the exogenous cell microenvironment or inner signaling pathways. A robustness index, *R*, was calculated for perturbations of the initial levels of phenotype-inducing microenvironments: M1 (LPS and IFNG-*γ*), M2a (IL-4), M2b (LPS and IL-1*β*) and M2c (IL-10). It may be observed that up to perturbation amplitudes *ϵ_R_*∼ 20%, the phenotype differentiation processes show a strong robustness with respect to initial-condition variations. In the case with *ϵ_R_*∼ 30%, only the robustness of the M2b phenotype is significantly reduced (**Table 3**). A similar analysis performed for the *β* parameter indicated that up to *ϵ_R_*∼ 30%, the robustness index *R* ≥ 0.98 in all the above mentioned cases. As an alternative stability proof, we translated the deterministic model into a stochastic scheme (**Figure 1** and 2.3.2) to explore the effect of intrinsic noise on the system dynamics (13, 28). Different levels of noise were introduced into the equations as described in Section 2.3.2, and 1,000 iterations were performed to obtain an average percentage of differentiated cells under each tested condition. **Figure 10** shows differentiation efficiencies (*ϵ_D_*) under M1, M2a, M2b and M2c input conditions, considering 0% to 50% noise. It can be seen that the differentiation process of all phenotypes is very robust, with *ϵ_D_* ≥ 0.9 (90%), except for M2c cells for noise levels higher than 30 %, where *ϵ_D_* exhibits a drastic reduction.

**Table 3:** Robustness index, *R*, for perturbation of initial levels of phenotype-inducing microenvironments: M1 (LPS and IFNG-*γ*), M2a (IL-4), M2b (LPS and IL-1*β*) and M2c (IL-10).

*R*				
*ϵ_R_* (%)	M1	M2a	M2b	M2c
10	0.98	0.98	0.99	0.98
20	0.96	0.97	0.97	0.96
30	0.89	0.95	0.66	0.95

It may be observed that up to perturbation amplitudes *ϵ_R_*∼ 20%, the phenotype differentiation processes shows a strong robustness with respect to initial-condition variations. In the case with *ϵ_R_*∼ 30%, only the M2b phenotype is significantly affected.

## 4 Discussion

Studies using single-cell RNA sequencing of monocytes and macrophages from different organs in physiological and pathological states (58) demonstrate that macrophage functional states are very diverse (**Figure 1**). The present model attempts to approach this notion, integrating signaling pathways from a number of surface receptors. The network topology highlights the significant convergence toward NF*κ*B from various independently acting elements, a characteristic reflected in the NF*κ*B Boolean rule (**Table 2**). Other transcription factors such as AP-1, CREB1, IRF3, IRF4, and IRF7 have more specific activation requirements and can act in concert with NF*κ*B and AP-1 to amplify the inflammatory response (18, 59, 60).

The anti-inflammatory role of CREB1 through the inhibition of NF-*κ*B was incorporated in the model, as well as the constitutive expression of the CREB1 inhibitor GSK3*β*. The model simulated the pathway of inhibition of CREB1 by IFN-*γ*, which involves four consecutive inhibitory interactions (see Section 2.3) and allowed to assess the role of IFN-γ and the requirement of GSK3β activity in the potentiation of the NF*κ*B-mediated inflammatory response (32). The transcription factor regulator TRAF3 is also a convergence point for signals initiated by Toll-like receptors. Likewise, the Syk kinase is a critical component where multiple signaling pathways converge, particularly those derived from phagocytosis receptors. In addition, the AMPK module allows the simulation of the pivotal role of metabolism in the phenotypic modulation of macrophages. Thus, the model formally exemplifies how key regulatory elements serve as hubs for the convergence of intricate signals during cellular responses.

The network dynamics reproduced the role of IL-4 and IL-10 in macrophage differentiation towards the M2a and M2b phenotypes through pathways converging in NF-*κ*B inhibition (**Figure 6**). So, the network successfully integrated signals modulating the activity of NF-*κ*B (61, 62). Additional nodes regulating NF-*κ*B may be incorporated in the model to fully capture its intricate function (work in progress).

Simulations allowed the integration of the TLR7 activation by ssRNA with the pro-inflammatory effect of IFN-*γ*, leading to a robust M1 differentiation, similar to that induced by the TLR4-LPS combination; IgG immune complexes (IC) added phagocytosis to the whole response (**Figure 8C**). The mathematical model may support the interpretation of experimental results regarding the effect of ssRNA, particularly when additional stimulus, as phorbol 12-myristate 13-acetate (PMA), a strong stimulator of PKC (and thus, of NF-*κ*B), are used to promote macrophage differentiation (63). The model predicts that stimulation by ssRNA in the absence of PMA would induce a transient production of TNF-*α* and IL-1*β* due to transient NF-*κ*B and IRF7 activities. Induction of stable expression of pro-inflammatory cytokines by ssRNA can be achieved in the presence of IFN-*γ* in the absence of PMA (this result can be achieved using the interactive program *Final monocyte network.ipynb* in the *Google Colab* web site).

Robustness analysis support the confidence of the model results and suggest that the macrophage functional network topology exhibits a strong stability under microenvironment and endogenous perturbations (∼ 90% for noise levels *Q* = 20%). In contrast, a similar analysis for the differentiation efficiency of a CD4 T-cells network exhibited a greater sensitivity to noise (∼ 60%, for *Q* = 20%) (28). So, our modeling strongly suggests that the network topology of intracellular macrophage signaling involves a structure less sensitive to random variations than the one of lymphocytes, perhaps due to natural selection mechanisms.

In summary, the mathematical model put forth here manages to reproduce the macrophage properties in varied initial conditions, underscoring their functional complexity and adaptability to the microenvironment. It turns out that the development of fully-polarized phenotypes arise under specific microenvironmental conditions. In general, diverse combinations and levels of exogenous agents lead to mixed-polarized expressions (M1/M2) (**Figure 7**), including modifications of metabolic processes. This is in line with the proposal of Palma et al. (9) concerning the possibility of a continuum of macrophage polarization transitions among M1 and M2 subtypes.

### 4.1 Implications and future directions

The model captures the dynamics of macrophage functional polarization in agreement with experimental observations, providing a solid framework for the comprehensive description of monocyte behavior. Further work should include interactions improving the prediction of functional features, like those driven by metabolic changes mediated by mTORC2, cytoskeletal dynamics and chemokine production. Other relevant functions of GSK3*β* in addition to inhibition of CREB1 (46) may be considered. The stimulatory effect of NF-*κ*B on the expression of phagocytosis receptors different from Fc*γ*R, like Dectin and the mannose receptor are incorporated in the model; mathematical simulation of signaling from them is a work in progress.

The modular structure of the monocyte/macrophage network allows its integration with regulatory networks pertaining to other cells of the immune system, or to tissues interacting with them through soluble factors. Work in progress involves connecting the present macrophage network with a CD4 T cell lymphocyte differentiation network (26) with the aim of simulating the immune response to respiratory infections (64), and that of diseases with chronic inflammatory origin, such as type 2 diabetes (25).

## Data Availability

The raw data supporting the conclusions of this article will be made available by the authors, without undue reservation.
