# Supplementary material for: An integrative model of pro- and anti-inflammatory signaling pathways in macrophage differentiation: the role of NF-κB and CREB
Source: Front Immunol. 2026 Jan 2;16:1639005. doi: 10.3389/fimmu.2025.1639005 (PMC12808404; doi:10.3389/fimmu.2025.1639005)
Supplement: Supplementary file 1 [file DataSheet1.pdf]

Supplementary Table 1: DOI Metadata.

Formatted Reference	DOI
K C Wilson and D S Finbloom, 1992	10.1073/pnas.89.24.11964
Timothy R. Hercus et al., 2012	10.3109/08977194.2011.649919
Charles A. Dinarello, 2009	10.1146/annurev.immunol.021908.132612
Mariana Romão-Veiga et al., 2020	10.1016/j.preghy.2020.06.002
Jérémy Loyau et al., 2014	10.4161/19420862.2014.975098
Carlos Rosales, 2017	10.3389/fimmu.2017.00280
Yasunobu Miyake et al., 2013	10.1016/j.immuni.2013.03.010
Bonder et al., 1999	10.1046/j.1365-2567.1999.00711.x
Eryn Bugbee et al., 2023	10.3389/fimmu.2023.1188750
Xiaoyi Hu et al., 2021	10.1038/s41392-021-00791-1
Piotr Przanowski et al., 2013	10.1007/s00109-013-1090-5
Alessandro Palma et al., 2018	10.3389/fphys.2018.01659
Xiaoyi Hu et al., 2021	10.1038/s41392-021-00791-1
Alessandro Palma et al., 2018	10.3389/fphys.2018.01659
Charles A. Dinarello, 2011	10.1182/blood-2010-07-273417
Sankar Ghosh et al., 1998	10.1146/annurev.immunol.16.1.225
Nolwenn Coudronniere et al., 2000	10.1073/pnas.97.7.3394
Olaf Gross et al., 2006	10.1038/nature04926
Osman Nidai Ozes et al., 1999	10.1038/43466
SANG-WOO KIM et al., 2014	10.3892/ijo.2014.2578
Guoqing Chen and David V. Goeddel, 2002	10.1126/science.1071924
Justin I. Odegaard et al., 2007	10.1038/nature05894
Alessandro Palma et al., 2018	10.3389/fphys.2018.01659
Fred D. Finkelman et al., 2000	10.4049/jimmunol.164.5.2303
Takashi Satoh et al., 2010	10.1038/ni.1920
Jacqueline D. Peda et al., 2016	10.1242/dmm.024745
Kevin W. Moore et al., 2001	10.1146/annurev.immunol.19.1.683
Katherine A. Fitzgerald et al., 2003	10.1038/ni921
Falk Nimmerjahn and Jeffrey V. Ravetch, 2008	10.1038/nri2206
Zhangguo Chen et al., 2003	10.1016/s0898-6568(03)00056-1
Xudong Liao et al., 2011	10.1172/JCI45444
Warren S. Alexander and Douglas J. Hilton, 2004	10.1146/annurev.immunol.22.091003.090312
Tianhao Duan et al., 2022	10.3389/fimmu.2022.812774
Takashi Satoh et al., 2010	10.1038/ni.1920
Mausumee Guha and Nigel Mackman, 2001	10.1016/s0898-6568(00)00149-2
Giorgio Trinchieri, 2003	10.1038/nri1001
Margarida Saraiva and Anne O'Garra, 2010	10.1038/nri2711
Nadra J Nilsen et al., 2008	10.1189/jlb.0907656
Mirjam Schenk et al., 2009	10.1016/j.immuni.2009.11.008
Saurabh Chattopadhyay and Ganes C. Sen, 2014	10.1089/jir.2014.0034
Jennifer M. Lund et al., 2004	10.1073/pnas.0400937101
Tiannan Hu et al., 2018	10.1016/j.bmc.2017.11.020
Ali Ashkar and Kenneth Rosenthal, 2002	10.2174/1566524023362159
Taro Kawai and Shizuo Akira, 2010	10.1038/ni.1863
Ruslan Medzhitov, 2001	10.1038/35100529
Isabelle Isnardi et al., 2008	10.1016/j.immuni.2008.09.015
Magdalena Koziczak-Holbro et al., 2007	10.1074/jbc.M700548200
Tatsukata Kawagoe et al., 2007	10.1084/jem.20061523
Milton Pereira and Ricardo T. Gazzinelli, 2023	10.3389/fimmu.2023.1133354
Yan-Ran Xu and Cao-Qi Lei, 2021	10.3389/fimmu.2020.608976
Zong-Ping Xia et al., 2009	10.1038/nature08247
Madoka Matsumoto-Ida et al., 2006	10.1152/ajpheart.00186.2005
Ana Cuenda and Simon Rousseau, 2007	10.1016/j.bbamcr.2007.03.010
J. Simon C. Arthur and Steven C. Ley, 2013	10.1038/nri3495
Yan-Ran Xu and Cao-Qi Lei, 2021	10.3389/fimmu.2020.608976
James P. Luyendyk et al., 2008	10.4049/jimmunol.180.6.4218
Roger J Davis, 2000	10.1016/s0092-8674(00)00116-1
Myung Soo Ko et al., 2022	10.1016/j.jbc.2022.101864
Paula Monje et al., 2005	10.1074/jbc.C500353200
Beatriz L. Caputto et al., 2014	10.1016/j.bbalip.2014.05.007
E. Minet et al., 2001	10.1006/excr.2001.5180
Atsuhiro Kanayama et al., 2004	10.1016/j.molcel.2004.08.008
Nicole Cusson-Hermance et al., 2005	10.1074/jbc.M506831200
Young-Hoon Park et al., 2014	10.1016/j.bbrc.2013.12.068
Kathryn C.S. Locker et al., 2022	10.2337/db21-0426
Yujuan Chen et al., 2021	10.1631/jzus.B2000808
Nicole Cusson-Hermance et al., 2005	10.1074/jbc.M506831200
Bipandeep Dhillon et al., 2019	10.3389/fimmu.2019.00104
Joanne M. Hildebrand et al., 2011	10.1111/j.1600-065X.2011.01055.x
Suzanne Paz et al., 2011	10.1038/cr.2011.2

Continued on next page

Supplementary Table 1: DOI Metadata (continued)

Formatted Reference	DOI
Takumi Kawasaki and Taro Kawai, 2014	10.3389/fimmu.2014.00461
Hans Häcker et al., 2011	10.1038/nri2998
Ke-Jun Han et al., 2004	10.1074/jbc.M311629200
Pradeep Bista et al., 2010	10.1074/jbc.M109.076091
Ajay Jain et al., 2014	10.3389/fimmu.2014.00553
Rui-Peng Wang et al., 2008	10.1016/j.molimm.2007.10.034
Tiejun Zhao et al., 2007	10.1038/ni1465
A. R. Brasier, 2010	10.1093/cvr/cvq076
Eric S. Hungness et al., 2000	10.1097/00024382-200014030-00025
Andy Y. Wen et al., 2010	10.4049/jimmunol.1001829
Andy Y. Wen et al., 2010	10.4049/jimmunol.1001829
Jia Zhou et al., 2014	10.1016/j.cyto.2014.05.003
Inna S Afonina et al., 2017	10.1038/ni.3772
Marcus V. Andrade et al., 2011	10.1002/eji.201040718
Mitsuharu Sato et al., 2000	10.1016/s1074-7613(00)00053-4
Rui-Peng Wang et al., 2008	10.1016/j.molimm.2007.10.034
Pablo Valverde et al., 2019	10.1021/acschembio.9b00458
Ting Liu et al., 2017	10.1038/sigtrans.2017.23
Alvin L. Smith et al., 2007	10.1084/jem.20061604
Tan Li et al., 2024	10.1080/16078454.2024.2330285
Elizabeth M. Terrell and Deborah K. Morrison, 2018	10.1101/cshperspect.a033746
Ranferi Ocaña-Guzman et al., 2023	10.1155/2023/3577334
Daniel Humphreys et al., 2016	10.1016/j.celrep.2016.09.039
Athanassios Dovas and Dianne Cox, 2010	10.4161/cib.3.2.10759
Edgar Pick, 2014	10.4161/sgtp.27952
Stephen Chiu and Ankit Bharat, 2016	10.1097/MOT.0000000000000313
Gwendalyn J Randolph et al., 2008	10.1016/j.coi.2007.10.010
Janice S. Blum et al., 2013	10.1146/annurev-immunol-032712-095910
Janice S. Blum et al., 2013	10.1146/annurev-immunol-032712-095910
Emmanuel C. Patin et al., 2017	10.3389/fimmu.2017.00861
Chunjie Li et al., 2020	10.1158/2326-6066.CIR-19-0782
Marc Daëron, 1997	10.1146/annurev.immunol.15.1.203
William J. Bradshaw et al., 2024	10.1016/j.str.2024.09.024
O. El-Hillal et al., 1997	10.1073/pnas.94.5.1919
Siaw Wei Ng et al., 2008	10.1074/jbc.M804942200
Wei Zou et al., 2008	10.1016/j.molcel.2008.06.023
Manit Munshi et al., 2022	10.1182/bloodadvances.2021006147
Ankit Malik et al., 2018	10.1016/j.immuni.2018.08.024
Marcel Deckert et al., 1996	10.1016/s1074-7613(00)80273-3
Vidya Vedham et al., 2005	10.1128/MCB.25.10.4211-4220.2005
Luana Caroline Oliveira et al., 2019	10.3389/fimmu.2019.02585
Rajesh K. Singh et al., 2019	10.1161/ATVBAHA.118.312087
Clelia Amato et al., 2019	10.1016/j.cub.2019.10.036
Alexis Bonfim-Melo et al., 2018	10.3389/fmicb.2018.00360
Wu and Pei, 2020	10.18632/aging.20492
Sonja I Gringhuis et al., 2009	10.1038/ni.1692
Helen S. Goodridge et al., 2007	10.4049/jimmunol.178.5.3107
C. A. Lowell, 2010	10.1101/cshperspect.a002352
C. A. Lowell, 2010	10.1101/cshperspect.a002352
Hendrik J. P. van der Zande et al., 2021	10.3389/fimmu.2021.765034
Hendrik J. P. van der Zande et al., 2021	10.3389/fimmu.2021.765034
Teunis B. H. Geijtenbeek and Sonja I. Gringhuis, 2016	10.1038/nri.2016.55
Ting Liu et al., 2017	10.1038/sigtrans.2017.23
Brian O. Smith et al., 2002	10.1016/s0092-8674(02)00672-4
Cássio Marinho Campelo et al., 2020	10.1016/j.exppara.2020.107970
Patrizia Scapini et al., 2009	10.1111/j.1600-065X.2008.00758.x
Patrizia Scapini et al., 2009	10.1111/j.1600-065X.2008.00758.x
H.S. Kim et al., 2012	10.1016/j.cellsig.2012.02.014
Olivia Hatton et al., 2011	10.1074/jbc.M111.255125
Masamichi Ishiai et al., 1999	10.1016/s1074-7613(00)80012-6
Youbang Xie et al., 2018	10.3892/mmr.2018.9713
RM Stone et al., 1988	10.1182/blood.V72.2.739.739
T Ueda et al., 1994	10.1073/pnas.91.22.10680
Yun-Long Zhang et al., 2024	10.1016/j.jare.2023.02.010
Amitava Mukherjee et al., 2023	10.1038/s41420-023-01538-3
Xinrui Li and Robert P Kimberly, 2014	10.1517/14728222.2014.877891
Huan-ping Zhang et al., 2024	10.1007/s12282-024-01567-5
Mathieu Laplante and David M. Sabatini, 2009	10.1242/jcs.051011

Continued on next page

Supplementary Table 1: DOI Metadata (continued)

**Formatted Reference**	**DOI**
Yang Zhao et al., 2023	10.1016/j.isci.2023.107540
Eun-Kyeong Jo et al., 2019	10.3389/fmicb.2019.00520
Duygu Sag et al., 2008	10.4049/jimmunol.181.12.8633
Eun-Kyeong Jo et al., 2019	10.3389/fmicb.2019.00520
Duygu Sag et al., 2008	10.4049/jimmunol.181.12.8633
Hiroaki Suzuki et al., 2016	10.1016/j.imlet.2016.05.009
Rebecca C. Rabinovitch et al., 2017	10.1016/j.celrep.2017.09.026
D Grahame Hardie, 2011	10.3945/ajcn.110.001925
Gayatri Devraj et al., 2017	10.1016/j.micinf.2016.11.003
Yao Tian et al., 2023	10.1038/s41523-023-00598-z
Takeshi Saito and Michael Gale, 2008	10.1084/jem.20081210
Daniel T. Thoresen et al., 2023	10.1016/j.molcel.2022.11.018
Bin Wu and Sun Hur, 2015	10.1016/j.coviro.2015.04.004
Run Fang et al., 2017	10.1371/journal.ppat.1006720
Hailing Hsu et al., 1995	10.1016/0092-8674(95)90070-5
